# NTSM: a non-salient target segmentation model for oral mucosal diseases

**DOI:** 10.1186/s12903-024-04193-x

**Published:** 2024-05-03

**Authors:** Jianguo Ju, Qian Zhang, Ziyu Guan, Xuemin Shen, Zhengyu Shen, Pengfei Xu

**Affiliations:** 1 School of Information Science and Technology, Northwest University, No.1, Xuefu Road, Xi’an, 710119 Shaanxi China; 2 Department of Oral Mucosal Diseases, Shanghai Ninth People’s Hospital, Shanghai Jiao Tong University School of Medicine, No.639, Manufacturing Bureau Road, HuangpuShanghai, 200011 China; 3 Department of Dermatology, Shanghai Ninth People’s Hospital, Shanghai Jiao Tong University School of Medicine, No.639, Manufacturing Bureau Road, HuangpuShanghai, 200011 China

**Keywords:** Oral mucosal diseases, Medical image segmentation, Convolutional neural network, Depthwise separable convolution, Non-salient target

## Abstract

**Background:**

Oral mucosal diseases are similar to the surrounding normal tissues, *i.e.,* their many non-salient features, which poses a challenge for accurate segmentation lesions. Additionally, high-precision large models generate too many parameters, which puts pressure on storage and makes it difficult to deploy on portable devices.

**Methods:**

To address these issues, we design a non-salient target segmentation model (NTSM) to improve segmentation performance while reducing the number of parameters. The NTSM includes a difference association (DA) module and multiple feature hierarchy pyramid attention (FHPA) modules. The DA module enhances feature differences at different levels to learn local context information and extend the segmentation mask to potentially similar areas. It also learns logical semantic relationship information through different receptive fields to determine the actual lesions and further elevates the segmentation performance of non-salient lesions. The FHPA module extracts pathological information from different views by performing the hadamard product attention (HPA) operation on input features, which reduces the number of parameters.

**Results:**

The experimental results on the oral mucosal diseases (OMD) dataset and international skin imaging collaboration (ISIC) dataset demonstrate that our model outperforms existing state-of-the-art methods. Compared with the nnU-Net backbone, our model has 43.20% fewer parameters while still achieving a 3.14% increase in the Dice score.

**Conclusions:**

Our model has high segmentation accuracy on non-salient areas of oral mucosal diseases and can effectively reduce resource consumption.

## Background

Oral mucosal diseases are functional injuries that occur in the tissue lining the mouth. These injuries often lead to abnormal growth of the tissue and increase the risk of cancer [1]. Early diagnosis and treatment of oral mucosal diseases have become important measures to prevent oral cancer. In current clinical practice, diagnosing oral mucosal diseases traditionally involves doctors observing the patient's mouth, which can be time-consuming and inconvenient for patients seeking treatment. It can be challenging for doctors to distinguish between normal oral tissue and mucosal lesions, making manual diagnosis difficult. To alleviate doctors from this tedious clinical load, automatic oral mucosal lesions segmentations are needed. This will not only benefit doctors but also patients seeking diagnosis and treatment.

Although deep learning algorithms have been widely used in medical image segmentation, they have rarely been carried out in oral diseases. Jubair et al*.* [2] proposed a lightweight transfer learning model to predict oral cancer based on convolutional neural networks (CNNs). Paderno et al*.* [3] performed semantic segmentation of oral cancer through a fully convolutional neural network, and the inference time of this method is particularly short, showing the possibility of real-time application. Both studies focused on identifying and segmenting oral cancer, but the extracted features are not accurate enough in location. Other studies, such as those by Farhad Ghazvinian Zanjani et al*.* [4] and Zhu et al*.* [5], focused on instance segmentation of teeth and caries, respectively. However, none of these methods considered differential feature learning in similar regions, resulting in unsatisfactory model performance. Some studies [6, 7] directly deployed commonly used segmentation models to the oral mucosal diseases dataset, but they did not fully consider the uniqueness of oral lesions, leading to poor segmentation performance and efficiency.

**Fig. 1:**
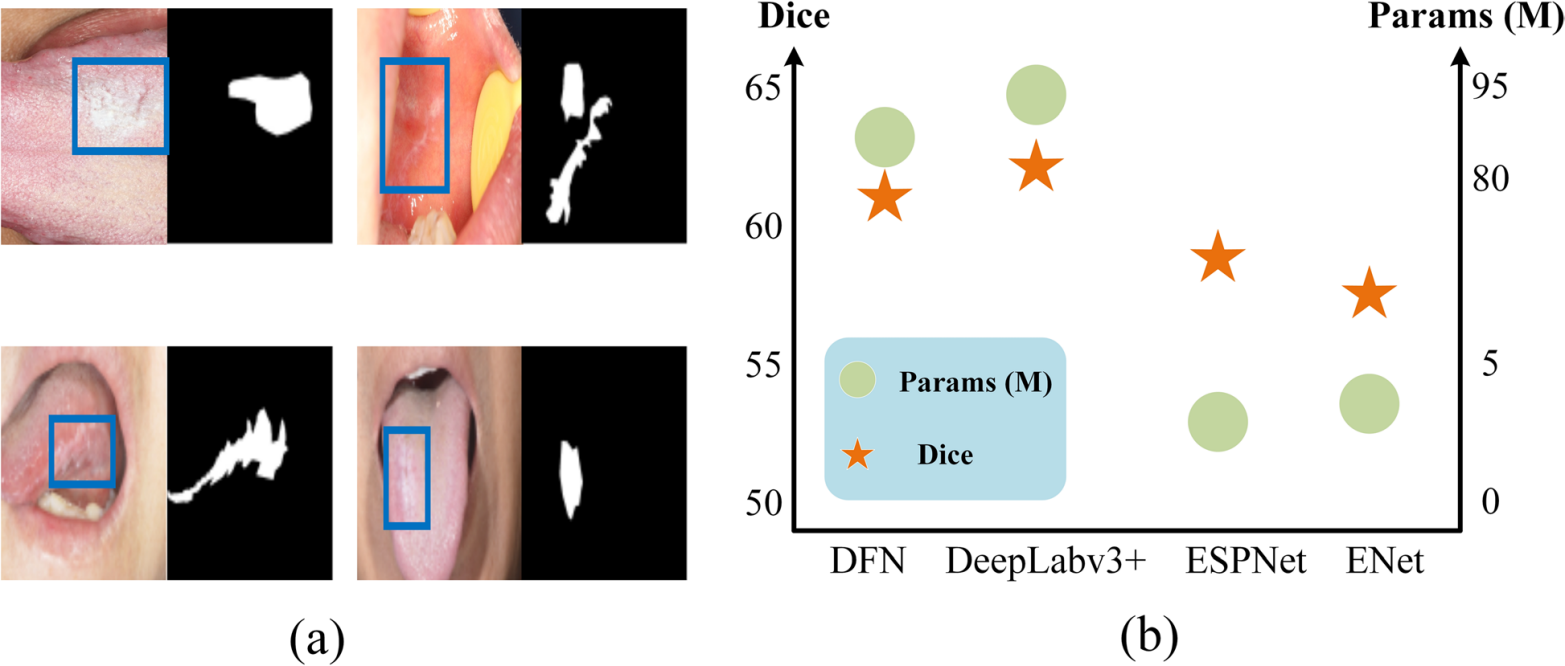
**a** Oral mucosal diseases closely resemble surrounding tissues. **b** Comparison of DFN, DeepLabv3 + , ESPNet, and ENet parameters

These above methods exploit the application of deep learning in oral mucosal diseases and provide a certain theoretical and practical foundation. However, there are still unconsidered challenges for automatic segmentation of oral mucosal diseases. On the one hand, as shown in Fig. 1 (a), the segmentation of oral mucosal diseases presents a challenge due to the similarity of these lesions with the surrounding tissue. This leads to a large number of non-prominent regions in oral mucosal lesions, which traditional automated segmentations cannot effectively handle. Similar challenges have been observed in natural image semantic segmentation. To address this, Mondal et al*.* [8] integrated multiple manually annotated features to discover and track nonsalient objects. However, these methods are limited in their expression ability and often fail to achieve satisfactory results. Researchers have developed other deep learning-based methods to address these issue and achieved good performance in non-salient object detection. Li et al*.* [9] applied joint learning to salient and non-salient feature tasks to balance local and contextual information. However, it did not fully consider the impact of logical semantic features, resulting in poor final results. Yan et al*.* [10] proposed using both instance segmentation and adversarial attacks to achieve camouflage object segmentation, which can effectively capture different scene layouts and improve segmentation performance. However, the expression ability of low-level features was not strong, which affected the segmentation effect [11, 12] proposed boundary recognition models and uncertainty models, extracting auxiliary information from shared contexts to analyze the feature differences between similar objects and their surrounding environment. However, these models tend to lose important local information on high-resolution images during the downsampling process. Ju et al*.* [13] presented a novel coarse-to-fine framework based on spatial contextual cues and active localization offset, which greatly solved the problem of difficult small organ segmentation, but did not fully consider the advantages of active learning theory. Pang et al*.* [14] proposed a mixed-scale ZoomNet for capturing objects in complex scenes at different "scaling" scales. The segmentation performance was some improvement, but the number of parameters and computations were too large. Ruozhen He et al*.* [15] proposed a CRnet based on structural information and semantic relationships, effectively utilizing low-level and high-level features to improve segmentation performance. However, it was not suitable for the needs of portable medical devices.

On the other hand, as shown in Fig. 1 (b), there are models like DFN [16] and DeepLabv3 + [17] that offer high accuracy in image segmentation, but they have too many parameters to be used on mobile devices. Models like ESPNet [18] and ENet [19] use depthwise separable convolution [20] to save computational memory, but they overlook the inherent attributes of image segmentation, leading to lower segmentation accuracy. Hence, segmentation models must balance performance and parameters. FDDWNet [21] used depthwise separable convolution to learn feature representations from different scale receptive fields with fewer model parameters. However, it neglected the inherent characteristics of semantic segmentation, resulting in poor segmentation accuracy. UTNet [22] was a simple and powerful hybrid Transformer architecture that integrated self-attention into convolutional neural networks for enhancing medical image segmentation, but the model computations were too high. UTNetV2 [23] was an improvement on UTNet that used depthwise separable convolution as a feedforward network for transformer blocks, reducing dependence on big data. However, it is still not suitable for portable mobile devices. Jeya et al*.* [24] combined MLP with U-Net, which proposed UNeXt, significantly reducing the number of parameters, but the visual effect of segmentation was average. Jiacheng Ruan et al*.* proposed a lightweight MALUNet [25], which achieved high skin lesions segmentation performance with lower parameters and computations. Moreover, EGE-UNet [26] was proposed in 2023, which was currently the first known model with a parameter count limit of 50KB.

In this paper, we propose a non-salient target segmentation model (NTSM) to achieve accurate segmentation of oral mucosal lesions with a smaller number of parameters. The NTSM includes a difference association (DA) module and a feature hierarchy pyramid attention (FHPA) module. The DA module has two submodules – the local context difference (LCD) and the logical semantic association (LSA) – which help extract local and contextual information, and semantic information, respectively. Continuously increasing the feature differences between the lesions and surrounding tissues in the oral cavity, improves the accuracy of segmentation of non-salient feature areas in oral mucosal diseases. To reduce the number of parameters in the model while maintaining high precision, we developed the FHPA module, which uses deep separable convolution for group learning. This approach allows us to achieve low-parameter yet high-precision segmentation of oral mucosal diseases. The main contribution can be summarized as follows:

- We design the NTSM model as a segmentation model for oral mucosal diseases. Our model joint action of the DA module and FHPA module achieves more accurate segmentation of non-salient lesions while minimizing the number of parameters, further inspiring the application of deep learning in the medical field.
- We develop a DA module that can learn local, contextual, and semantic information by using convolutional neural networks. The DA helps to increase the differences between real lesions and backgrounds, promoting segmentation performance for non-salient feature regions.
- The FHPA module we proposed realizes parameter sharing through depthwise separable convolution, effectively reducing the number of parameters and computations for high-precision large models, thereby decreasing the cost of model training and inference.
- We conduct a series of comparative experiments on the private dataset oral mucosal diseases (OMD) and the public dataset international skin imaging collaboration (ISIC) to verify the effectiveness and innovation of our model. The experimental results demonstrate that our model not only enhances segmentation accuracy but also reduces the number of parameters.

## Methods

### Datasets

We conduct for the study using two datasets, oral mucosal diseases (OMD) and international skin imaging collaboration (ISIC). The OMD dataset is a two-dimensional RGB oral image dataset collected from hospitals, consisting of 1051 original oral images in JPG format and corresponding segmentation masks in PNG format. Based on previous research experience, the training set consists of 812 images (including the validation set of 162 images), while the test set consists of 239 images. The ISIC dataset for skin diseases melanoma is publicly available, consisting of 2000 raw images in JPG format and corresponding segmentation masks in PNG format. We select 1800 images as the training set (including the validation set of 360 images) and the remaining 200 as the test set. Moreover, due to the pre-processing of our model having precise requirements on the input image format, we need to set the size of all images to 512 × 512 uniformly and generate the unique ID of the two datasets, respectively. Then, the JPG format of the original image is converted to the lossless PNG format. Finally, the segmentation mask is converted into a single-channel image with a pixel value of 0 or 1 and is added at the end of each file name with the '0000' symbol.

### Non-salient target segmentation model (NTSM)

**Fig. 2:**
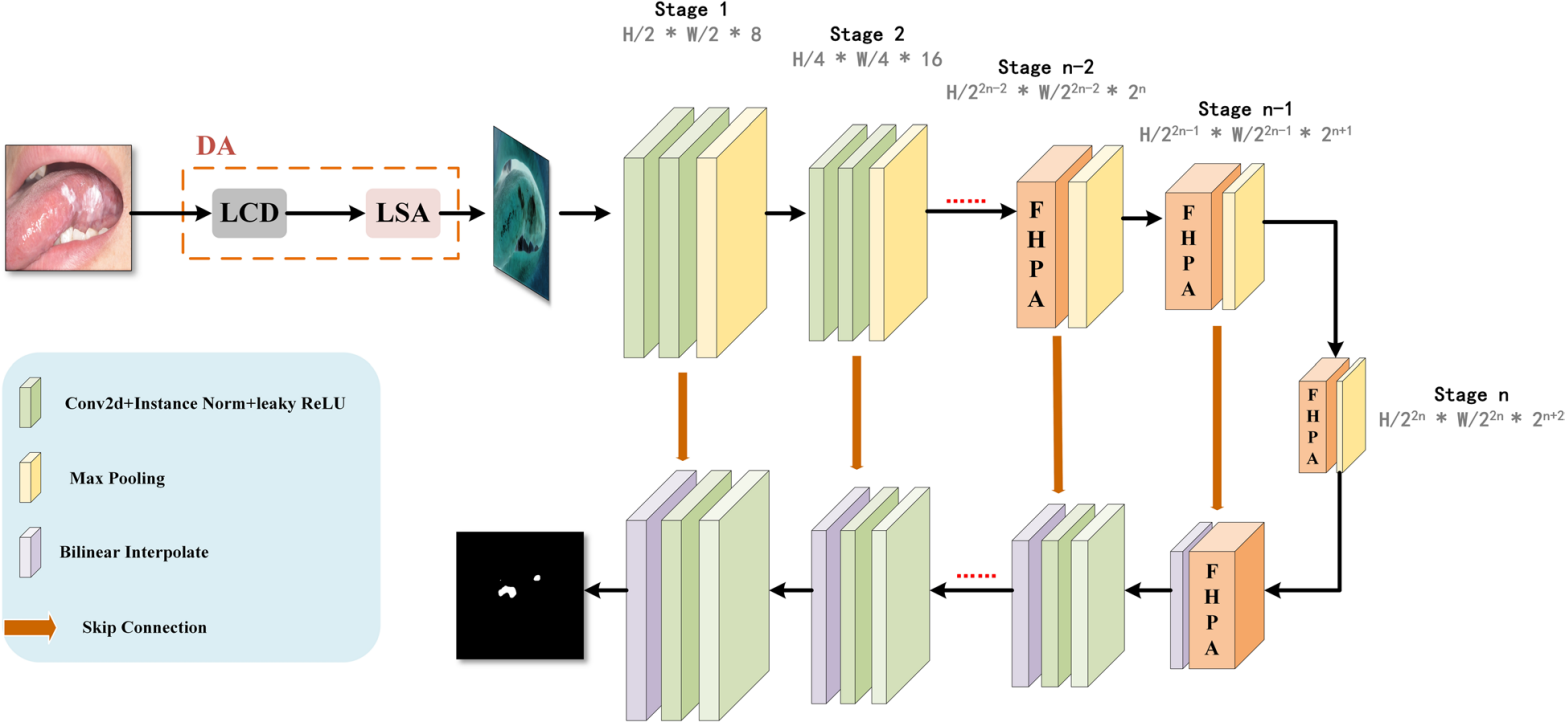
The overview of non-salient target segmentation model (NTSM). The original image is first processed by the difference association (DA) module. This module passes the image through two submodules: the local context difference (LCD) submodule and the logical semantic association (LSA) submodule. These submodules output feature difference maps, which are then passed into the encoder-decoder structure. The encoder-decoder structure outputs the corresponding feature maps in sequence. Additionally, the last three layers of the encoder and the first layer of the decoder use the feature hierarchy pyramid attention (FHPA) module instead of the regular convolutional layer to decrease the number of parameters

We present a non-salient target segmentation model (NTSM) to segment lesions of oral mucosal diseases from medical images. As shown in Fig. 2, we use nnU-Net as the backbone, and the main innovative modules are the difference association (DA) module and the feature hierarchy pyramid attention (FHPA) module. We design the DA module to continuously increase the feature differences between oral lesions and non-lesions at different levels, and then improve the segmentation accuracy of high-similarity regions. We design the FHPA module to extract oral feature information from different views through depthwise separable convolution, reducing the number of parameters while maintaining stable segmentation performance. We next detail the DA module and FHPA module as follows.

#### Difference association (DA) module

In oral medical images, the features of the lesions and surrounding tissues (such as intensity and texture) are relatively similar, *i.e.,* there are more non-salient lesions in oral mucosal diseases. Existing methods [6, 7, 11, 12] cannot effectively enhance the feature differences between lesions and non-lesions areas, resulting these methods in poor performance for lesions segmentation with non-salient features. Inspired by the visual inhibition mechanism on the mammalian retina [27], we propose a DA module to make the features of oral mucosal non-salient lesions more prominent. The DA includes a local context difference (LCD) submodule and a logical semantic association (LSA) submodule. The LCD sub-module increases the difference between low-level features by learning the local and contextual information of the lesions, while the LSA sub-module increases the difference between high-level features by learning the semantic logic information between the lesions and surrounding tissues. These two sub-modules work together to make the boundaries of oral lesions clearer and significantly improve segmentation performance.

##### Local context difference (LCD) submodule

**Fig. 3:**
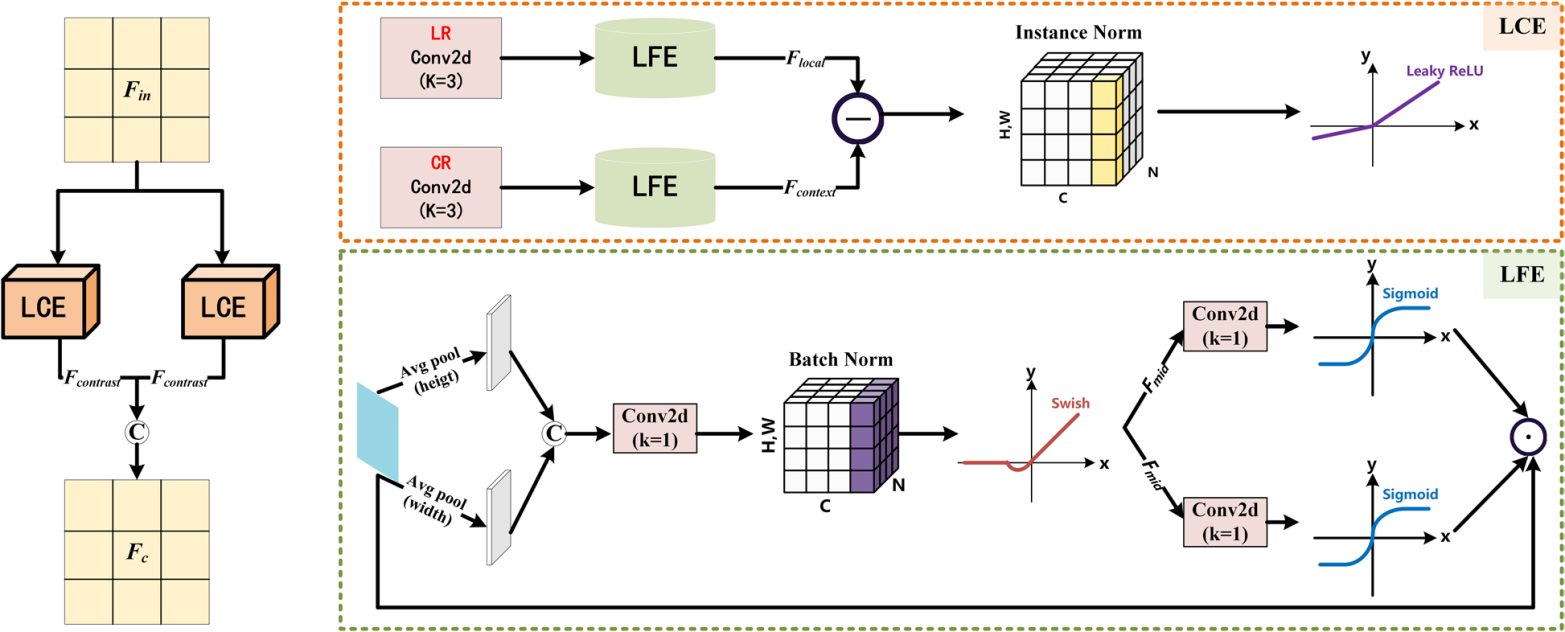
Illustration of LCD submodule. Depending on the receptive domain, a LCD submodule includes two low-level comparison extractors (LCE) and four low-level feature extractors (LFE). The diagram on the right shows the specific structure of LFE

In convolutional neural networks (CNNs), different convolution kernel sizes are used to extract various oral features [28]. However, a single-branch structure is inadequate for comprehensively extracting oral low-level features. To address this limitation, we propose a multi-branch LCD submodule to improve the identification of low-level oral features, which helps in learning local and contextual information about lesions. As shown in Fig. 3, the LCD submodule contains two low-level comparison extractors (LCE). The LCE is used to improve low-level contrast and clarity by calculating oral low-level comparison information. Each LCE consists of a local receptor (LR), a context receptor (CR), and two low-level feature extractors (LFE). The LFE uses cross-spatial and channel attention mechanisms to extract oral low-level features. This improvement helps to extend the segmentation mask to potentially similar regions, enabling our model to better discover the main structure and prospective boundaries of oral lesions.

In LCD execution processes, the input oral low-level features are firstly divided into four branches by different receptive fields (LR or CR) and entered into LCE to calculate the low-level comparison features of lesions. Then, the oral low-level features go through a 3 × 3 convolutional layer and LFE to extract local and contextual information. In each LFE operation, the input feature *F*_*in*_ is taken into a 1-dimensional horizontal and average pooling separately. After connecting the above results and then the intermediate feature *F*_*mid*_ is obtained through a 1 × 1 convolution layer, batch normalization, and swish activation function. We divide *F*_*mid*_ into ${{{F}}}_{{{m}}{{i}}{{d}}{ }}^{{{h}}}\in { }{{C}}{ }\times { }{{h}}{ }\times { }1$ and ${{{F}}}_{{{m}}{{i}}{{d}}{ }}^{{{w}}}\in { }{{C}}{ }\times { }1{ }\times { }{{w}}$, which through a 1 × 1 convolution layer and sigmoid function, respectively. Finally, the results of these two parts are multiplied by the features before average pooling, and obtain local feature *F*_*local*_ or contextual feature *F*_*context*_ of oral lesions. The mathematical expression is as follows:

1 $$\left\{ {\begin{array}{*{20}c} {F_{local} = O_{LFE} (F_{in} ,R_{LR}(f^{3 \times 3} ,d = 1))} \\ {F_{{conte{\text{x}}t}} = O_{LFE} (F_{in} ,R_{CR}(f^{3 \times 3} ,d = 4 \,or \,8))} \\ \end{array} } \right.$$

where *O*_*LFE*_ indicates performing low-level feature extractors operation. The *R*_*LR*_($\cdot$) represents a 3 × 3 convolutional layer with 1 dilation rate and the *R*_*CR*_($\cdot$) represents a 3 × 3 convolutional layer with 4 or 8 dilation rate. The purpose of setting different dilation rates is to extract low-level comparative features concentrated on different receptive fields, expanding the segmentation mask of oral lesions to potentially similar regions. Because instance normalization helps eliminate internal biases in the dataset, making the inputs of each layer in the network more consistent, thereby improving the training and inference performance of the model. Moreover, the Leaky ReLU compensates for the shortcomings of ReLU in negative intervals to solve the problem of gradient vanishing. Therefore, after *F*_*local*_ subtracting *F*_*context*_, it performs instance normalization and Leaky ReLU. *i.e.,* which calculates the low-level contrast feature *F*_*contrast*_ through LCE operation. The mathematical expression is as follows:

2 $$F_{contrast} = O_{LCE} (F_{local} - F_{{conte{\text{x}}t}} )$$

where *O*_*LCE*_ denotes performing low-level comparison extractors operation. Finally, through LCD operation and output ${{{F}}}_{{{c}}}={{{O}}}_{{{L}}{{C}}{{D}}}{ }({{{F}}}_{{{c}}{{o}}{{n}}{{t}}{{r}}{{a}}{{s}}{{t}}}$), which enables our model to better identify the main structure and potential boundaries of oral lesions. The algorithm flow is illustrated in Algorithm 1.

**Figure:**
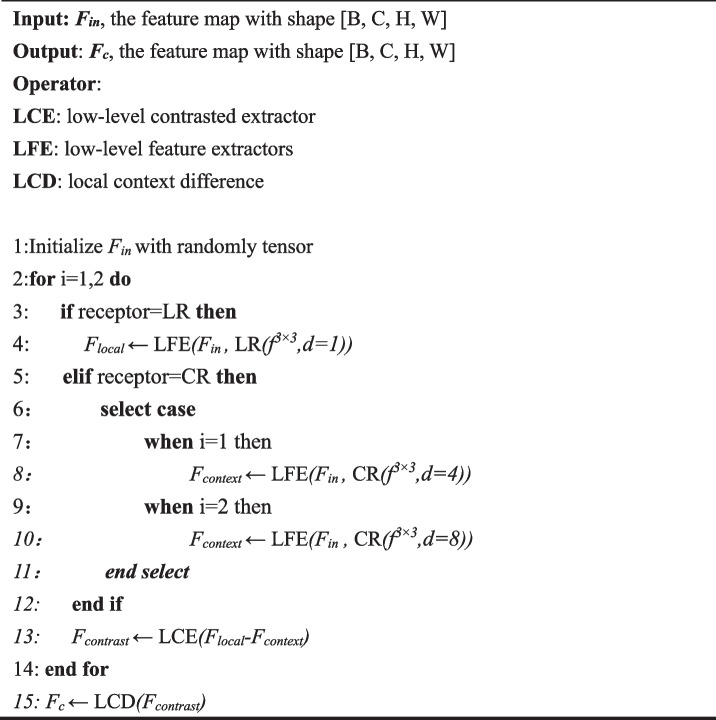
** Algorithm 1.** Local context difference (LCD) submodule

##### Logical semantic association (LSA) submodule

In the LCD, the submodule learned the low-level features that can be extracted directly from the oral data set, such as color, shape, and other information. However, the submodule did not consider the impact of deeper high-level features, such as semantic relationships, on segmentation performance. For this reason, when the background is composed of many high-level contrastive parts (such as oral lichen planus and teeth), we design the LSA submodule to extract logical semantic relationship information from branches of different receptive fields to identify the true foreground and background of oral lesions accurately.

**Fig. 4:**
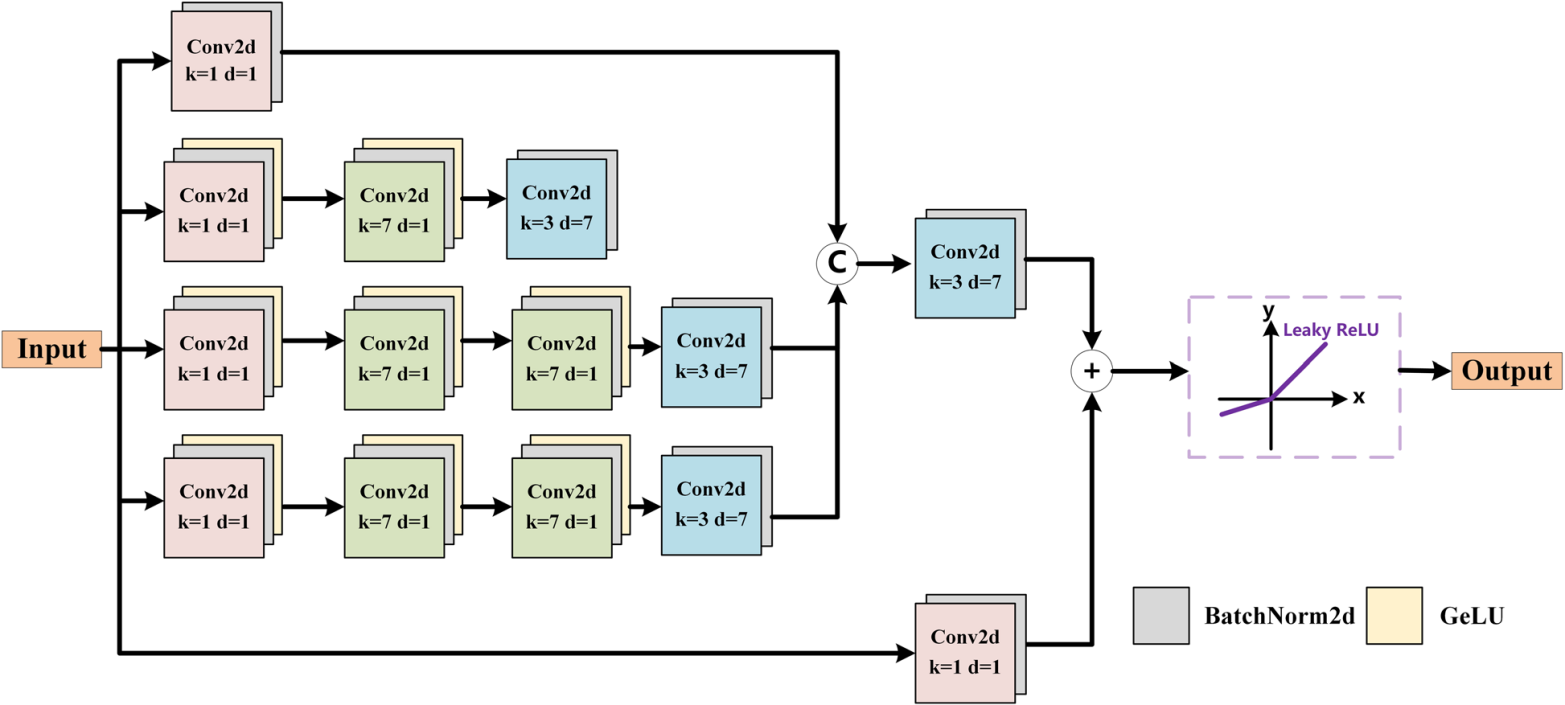
Illustration of LSA submodule. Learning different logical semantic information through convolutional layers with different kernel sizes and dilation rates

As shown in Fig. 4, we input feature *F*_*c*_ into four branches, each containing a series of convolution layers with different kernel sizes and dilation rates, representing different oral receptive fields. The first branch only goes through a 1 × 1 convolution layer to extract preliminary logic-semantic relationships of lesions. The second branch goes through a 1 × 1 convolution layer, a 7 × 7 convolution layer, and a 3 × 3 convolution layer. In the last convolution layer, the dilation rate set is 7 to obtain lesions features at different scales. The last two branches are successively passed through a 1 × 1 convolutional layer, two 7 × 7 convolutional layers, and a 3 × 3 convolutional layer. In the last convolutional layer, the dilation rate set is 7 to improve the accuracy of feature recognition in oral lesions. The mathematical expression can be expressed as:

3 $$\left\{ {\begin{array}{*{20}c} {F_{c}^{1} = {\text{BN}}(f^{1 \times 1} ,d = 1)} \\ {F_{c}^{2} = {\text{BN}}(f^{3 \times 3} ({\text{GelU}}({\text{BN}}(f^{7 \times 7} ({\text{GelU}}({\text{BN}}(f^{1 \times 1} ,d = 1))),d = 1))),d = 7)} \\ {F_{c}^{3} = F_{c}^{4} = {\text{BN}}(f^{3 \times 3} ({\text{GelU}}({\text{BN}}(f^{7 \times 7} ({\text{GelU}}({\text{BN}}(f^{7 \times 7} ({\text{GelU}}({\text{BN}}(f^{1 \times 1} ,d = 1))),d = 1))),d = 1))),d = 7)} \\ \end{array} } \right.$$

where BN($\cdot$) represents batch normalization. GeLU($\cdot$) is an activation function, which not only makes gradient calculation simpler, but also ensures better training stability of the oral model. In the four branches, only the last convolutional layer performs batch normalization, and each of the remaining convolutional layers will perform batch normalization and GeLU activation functions, so that the logical semantic features of different receptive fields can be combined better integration. Four branches are used to extract high-level semantic features at different scales and spatial resolutions, thereby enhancing the generalization ability of the oral segmentation model on new test sets. In addition, after connecting the output features of the four branches, use a 3 × 3 convolution layer and batch normalization for processing, and compare the above results with the results after using a 1 × 1 convolution layer and batch normalization. The output features are added and then passed through the Leaky ReLU activation function to enhance the nonlinear expression ability of the model and obtain the final output feature *F*_*s*_:

4 $$\left\{\begin{array}{c}{F}_{t}={F}_{c}^{1}\mathrm{\copyright }{F}_{c}^{2}\mathrm{\copyright }{F}_{c}^{3}\mathrm{\copyright }{F}_{c}^{4}\\ {F}_{s}=\mathrm{Leaky \,ReLU}(({\text{BN}}({f}^{3\times 3}({F}_{t}),d=7))\oplus ({\text{BN}}( {f}^{1\times 1},d=1)))\end{array}\right.$$

where © represents concatenation and $\oplus$ is addition. Finally, this submodule obtains comprehensive logical semantic features *F*_*s*_ through a wide range of receptive fields to determine the true oral lesions.

#### Feature hierarchy pyramid attention (FHPA) module

The DA module tackles the challenge of distinguishing oral lesions from surrounding tissues. However, to improve segmentation performance, we increase the model depth to learn complex pathological features in medical images. This leads to a large number of parameters, making it unsuitable for portable medical devices [29]. To address these issues, we propose a FHPA module that is evenly divided into four groups along the channel dimension. This module extracts oral feature information from multiple views, ensuring segmentation performance accuracy, while effectively reducing the number of parameters.

**Fig. 5:**
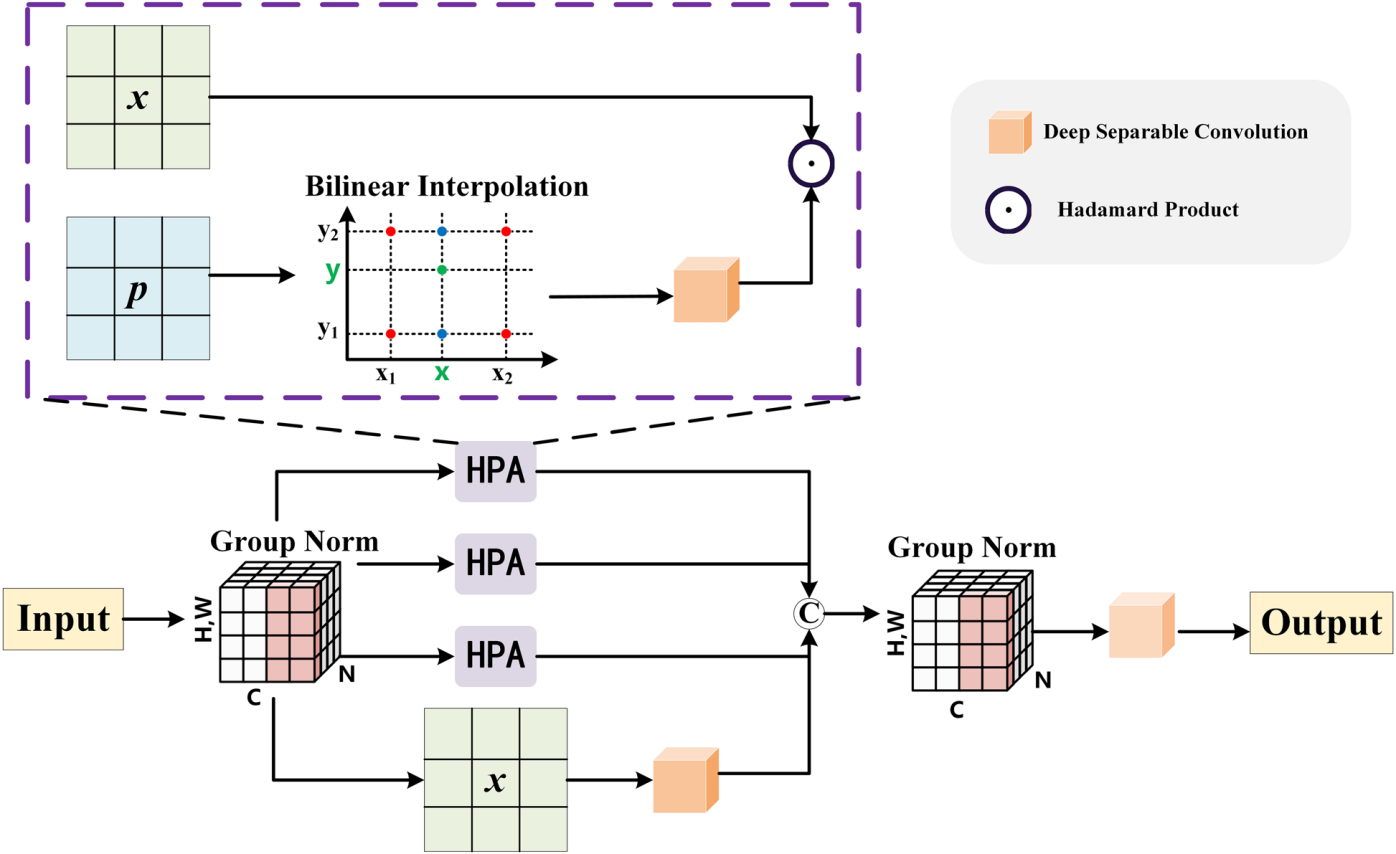
Illustration of FHPA module. The input features are evenly divided into four groups along the channel dimension, and multi-axis hadamard product attention (HPA) operations are performed in the first three groups to extract feature information from multiple views

In previous works, Jiang Ruand et al*.* [25] proposed a multi-axis hadamard product attention (HPA) with linear complexity. HPA takes an input feature *x* and a randomly initialized learnable tensor *p*. Then it adjusts the size of *p* to match *x* through bilinear interpolation (BI), preserving the spatial information in *x* better. Finally, it uses depthwise separable convolution (DW) for *p*, and performs hadamard product (HP) between *x* and *p* to obtain the output feature. The HPA can effectively extract and fuse multi-scale feature information from the input feature map *x*, which reduces the number of parameters. However, using a single HPA module can extremely decrease the segmentation performance. Our FHPA module addresses the aforementioned issues, and its calculation process is shown in Fig. 5. Initially, the input feature *x* uses group normalization, and the *CHUNK* function is employed to divide *x* into four groups evenly along the channel dimension. This enables the model to effectively extract feature information from oral images with different views. The mathematical expression for this process is as follows:

5 $$x1, x2, x3, x4 = CHUNK({\text{GN}}(x), 4, \dim = 1)$$

where GN($\cdot$) represents group normalization. The *x*_*1*_, x_2_, and *x*_*3*_ correspond to the features of the height-width (xy) axis, channel-height (zx) axis, and channel-width (zy) axis in oral images, respectively. After randomly initialized learnable tensors *p*_*xy*_, *p*_*zx*_, and *p*_*zy*_, each of these three groups performs HPA operation, and makes the model more flexible to integrate oral feature information at different scales. The mathematical expressions can be expressed as:

6 $$\left\{ {\begin{array}{*{20}c} {y_{1} = H_{DW} (H_{BI} (p_{xy} ))\odot x_{1} } \\ {y_{2} = H_{DW} (H_{BI} (p_{zx} ))\odot x_{2} } \\ {y_{3} = H_{DW} (H_{BI} (p_{zy} ))\odot x_{3} } \\ \end{array} } \right.$$

where $\odot$ indicates hadamard product operation, *H*_*DW*_ denotes depthwise separable convolution operation, and *H*_*BI*_ represent bilinear interpolation operation. The hadamard product can simplify the calculation process of attention weights and reduce the number of parameters and computations of the oral model. For the last set of *x*_*4*_, DW is used only on the oral feature map, *i.e.,*${{{y}}}_{4}={{{H}}}_{{{D}}{{W}}}({{{x}}}_{4})$, through a 1 × 1 convolution layer, the GeLU activation function, and a 3 × 3 convolution layer in turn. By depthwise separable convolution to realize parameter sharing, the number of computations is reduced, and the oral feature information is effectively extracted. In the end, the output feature of the above four groups is connected by the *CAT* function along the channel dimension, and group normalization is carried out. Then, another DW is used, *i.e.,* a 3 × 3 convolution layer, GeLU activation function, and a 1 × 1 convolution layer are used successively so that the model can re-integrate the feature information of color, shape, and boundary in oral images from different views. The mathematical expressions can be expressed as:

7 $$x=H_{DW}(\text{GN}(CAT(\lbrack y_1,y_2,y_3,y_4\rbrack,\dim=1)))$$

#### Loss function

We use deep supervision in our work to generate more accurate mask information for modules requiring different scales and views. We employ binary cross-entropy loss to measure the difference between predicted and ground truth, which helps the model learn accurate label probability distributions. Additionally, we use dice loss to measure the degree of overlap between the predicted and ground truth bounding boxes. The closer the value is to 1, the better the model's performance as it indicates more overlapping between the predicted and real regions. However, a single loss function is insufficient for multi-task learning, so we use a joint loss function to better guide model training and achieve optimal predictive performance. Our loss function can be expressed as follows:

8 $$L = \sum\limits_{i = 0}^{n} {\lambda_{i}^{{}} } \left( {Bce(y_{{}} ,\hat{\overline{y}}) + k \cdot Dice(y_{{}} ,\hat{\overline{y}})} \right)$$

where *k* represents hyperparameters and $\lambda_i$ means the weights of different stages.

### Evaluation metrics

During our study, we use sensitivity (Sen), specificity (Spe), dice similarity coefficient (Dice), and 95% hausdorff distance (95HD) to measure the segmentation performance of our model. The Sen refers to the probability of correctly identifying patients with the diseases, *i.e.,* the probability of not missing a diagnosis. The Spe estimates the probability of correctly identifying non-patients when the diseases is not present, *i.e.,* the probability of not being misdiagnosed. The Dice measures the model's accuracy by calculating the degree of overlap between the predicted results and the true labels. It is sensitive to the internal filling of the segmentation mask. The 95HD describes the similarity between two sets of point sets by representing the distance between them. It is sensitive to the segmented boundaries. Our model mainly enhances the feature boundaries of similar regions. Therefore, we comprehensively consider all four indicators to compare and analyze the segmentation performance. The mathematical expressions of the four evaluation indicators are as follows:

9 $$\left\{ {\begin{array}{*{20}c} {{\text{Sen}} = \frac{TP}{{TP + FN}}} \\ {{\text{Spe}} = \frac{TN}{{TN + FP}}} \\ \begin{gathered} {\text{Dice}} = \frac{2TP}{{FP + 2TP + FN}} \hfill \\ \hfill \\ \end{gathered} \\ {{\text{95HD}} = \max \left( {\mathop {\max }\limits_{a \in A} \left\{ {\left. {\mathop {\min }\limits_{b \in B} \left\| {a - b} \right\|} \right\}} \right.,\mathop {\max }\limits_{b \in B} \left\{ {\left. {\mathop {\min }\limits_{a \in A} \left\| {b - a} \right\|} \right\}} \right.} \right)} \\ \end{array} } \right.$$

where *TP*, *FP*, *FN*, and *TN* represent true positive, false positive, false negative, and true negative, respectively. *A* and *B* represent two sets of point sets, and *a* and *b* represent the corresponding elements in the point set. Moreover, we use Params (M), FLOPs (G), and Memory (M) to evaluate the segmentation efficiency of our model. Params (M) represents the number of parameters in the model, independent of the input data and related to the structure of the model. FLOPs (G) refer to the quantity of floating-point operations per second. Memory (M) represents the weight size of the trained model. We analyze the results of these indicators to determine whether our model is suitable for portable mobile devices.

## Results

### Implementation details

We use PyTorch for all our experiments and run them on a GeForce GPU RTX3080 device. During the training phase, we use the nnU-Net model as the backbone, with an initial learning rate of 0.001 to update the model's weight and bias. We set the weight attenuation to 0.00003 to prevent overfitting of the model. To address the problem of category imbalance, we set the ratio of lesions to background to 0.33. In each epoch, we use a batch size of 4, train the data for 250 iterations, validate the data for 250 iterations, and run a total of 300 training epochs. We conduct five cross-validation tests to reduce the impact of randomness on the experimental results.

### Comparison with state-of-the-art methods

We conduct comparative experiments between our model with state-of-the-art (SOTA) methods on the private dataset OMD and public dataset ISIC. The purpose is to validate the rationality of our proposed model and to enhance its credibility. These SOTA methods include: U-Net [30], Attention UNet [31], nnU-Net [32], UNeXt [24], MALUNet [25], CRnet [15], EGE-UNet [26], TransAttUNet [33], FCN-8 [34], Mask2Former [35], OneFormer [36].

The results of our segmentation analysis are presented in Table 1. We used sensitivity, specificity, 95HD, and Dice evaluation indicators to assess the performance of our model and SOTA methods. U-Net has a sensitivity of 40.51%, specificity of 98.93%, a 95HD of 23.16mm, and a Dice of 44.71%. nnU-Net has a sensitivity of 70.81%, specificity of 99.49%, a 95HD of 9.89mm, and a Dice of 73.72%. The EGE-UNet method, based on U-Net, has a sensitivity of 67.97%, specificity of 99.01%, a 95HD of 13.96mm, and a Dice of 65.61%. Compared to U-Net, EGE-UNet has an increase in sensitivity, specificity, and Dice by 27.46%, 0.08%, and 20.9% respectively, whereas the 95HD decreased by 9.2mm. All indicators for EGE-UNet are inferior to nnU-Net. It has been observed that U-Net and EGE-UNet models do not consider the impact of feature differences in non-salient lesions on segmentation performance. To improve the segmentation performance, our model is designed by incorporating a DA module that enhances non-salient feature differences. Our model is tested against the U-Net, EGE-UNet, and nnU-Net models, and it was found to outperform the other models in terms of sensitivity, specificity, and Dice score. Specifically, compared to U-Net, our model showed an increase of 30.49% in sensitivity, 0.63% in specificity, and 32.15% in Dice score, while 95HD decreased by 14.28mm. Compared to EGE-UNet, our model showed an increase of 3.03% in sensitivity, 0.55% in specificity, and 11.25% in Dice score, while 95HD decreased by 5.08mm. Finally, compared to nnU-Net, our model showed a slight increase of 0.19% in sensitivity, 0.07% in specificity, and 3.14% in Dice score, while 95HD decreased by 1.01mm. These results indicate that our model can better enhance feature differences at different levels, resulting in better segmentation performance than other methods in areas with high similarity and non-salient lesions.

**Table 1:** Comparative experimental results for segmentation performance on OMD and ISIC datasets

Datasets	OMD			
Model	Sen (%)↑	Spe (%)↑	Dice (%)↑	95HD (mm)↓
U-Net (2015) [30]	40.51	98.93	44.71	23.16
Attention UNet (2018) [31]	57.33	99.06	63.54	15.36
nnU-Net (2021) [32]	70.81	99.49	73.72	9.89
UNeXt (2022) [24]	66.44	99.28	63.44	10.18
MALUNet (2022) [25]	60.25	99.18	63.81	14.18
CRnet (2022) [15]	60.47	99.25	65.59	15.08
EGE-UNet (2023) [26]	67.97	99.01	65.61	13.96
TransAttUNet (2023) [33]	62.03	98.01	67.77	13.78
**NTSM (ours)**	**71.00**	**99.56**	**76.86**	**8.88**
**Datasets**	**ISIC**			
**Model**	**Sen (%)↑**	**Spe (%)↑**	**Dice (%)↑**	**95HD (mm)↓**
U-Net (2015) [30]	93.81	99.38	78.15	1.96
Attention UNet (2018) [31]	93.88	99.36	93.17	1.62
nnU-Net (2021) [32]	95.95	99.09	93.40	1.24
UNeXt (2022) [24]	87.77	99.15	89.77	1.42
MALUNet (2022) [25]	92.24	99.55	93.52	1.24
CRnet (2022) [15]	93.08	99.64	94.28	1.68
EGE-UNet (2023) [26]	93.04	99.40	93.01	1.61
TransAttUNet (2023) [33]	94.52	99.28	93.17	1.50
**NTSM (ours)**	**93.64**	**99.56**	**94.31**	**0.97**

**Fig. 6:**
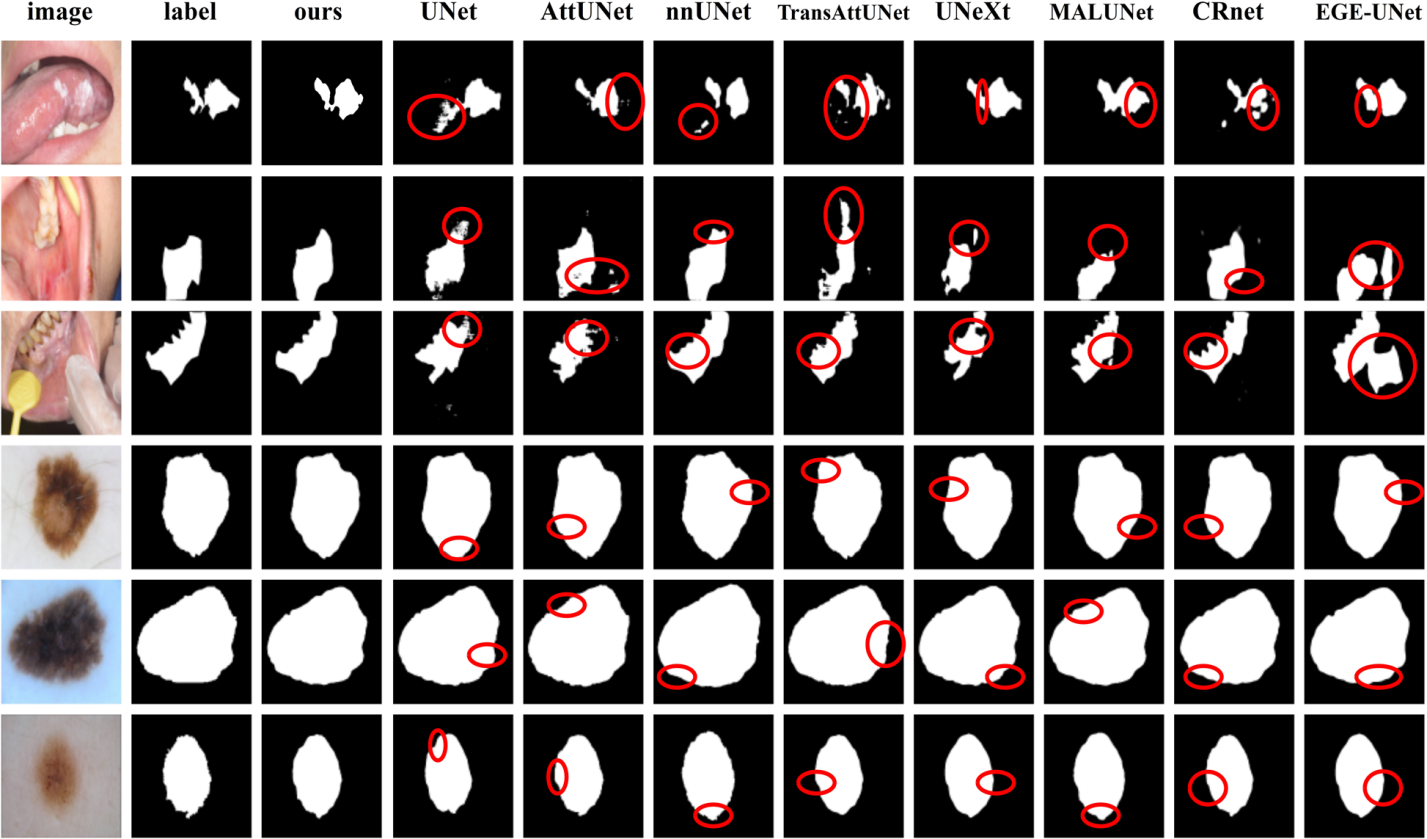
The segmentation results from different models. The first column displays the original image, while the second column shows the actual label of the image segment. The third column represents the segmentation result of our model, and columns 4–11 demonstrate the segmentation results of the SOTA methods

We visualize the segmentation results of our model compared to SOTA methods in Fig. 6. The EGE-UNet method performs better than U-Net regarding lesions segmentation, but still falls short of nnU-Net segmentation results. These methods lack proper feature learning in non-salient lesions. Our model, on the other hand, achieves more accurate segmentation results as it learns local, contextual, and logical semantic information, which helps in achieving finer segmentation boundaries and more complete regions, as shown in column 3.

Our model uses nnU-Net as the backbone, but this results in a large number of parameters. Therefore, our proposed model significantly reduces the number of parameters and computations while ensuring segmentation performance, but it’s not as lightweight as some other models. As shown in Table 2, the results obtained using Params (M), FLOPs (G), and Dice evaluation indicators are mainly used to analyze our and some large model's segmentation efficiency. Among them, the Params (M) of nnU-Net is 126.56M, FLOPs (G) is 466.23G, Memory (M) is 353.69M, and Dice is 73.72%; The Params (M) of Mask2Former is 215.23M, FLOPs (G) is 473.85G, Memory (M) is 826.02M, Dice is 63.04%. They all have a large number of parameters and computations. Our model designs an FHPA module that significantly reduces the number of parameters and computations while still maintaining better segmentation performance. When compared to nnU-Net, Params (M), FLOPs (G), and Memory (M) decrease by 54.68M, 44.51G, and 100.05M, respectively, while Dice increases by 3.14%. When compared to Mask2Former, our model's Params (M), FLOPs (G), and Memory (M) decrease by 143.35M, 52.13G, and 572.38M respectively, while Dice increases by 13.82%. These results indicate that our model can significantly reduce the number of parameters and computations by using depthwise separable convolution, while maintaining segmentation performance using feature learning.

**Table 2:** Comparative experimental results for segmentation efficiency on OMD and ISIC datasets

Model	Params (M)↓	FLOPs (G)↓	Memory (M)↓	OMD-Dice (%)↑	ISIC-Dice (%)↑
FCN-8 (2017) [34]	134.28	466.39	378.26	60.37	82.15
nnU-Net (2021) [32]	126.56	466.23	353.69	73.72	93.40
Mask2Former (2022) [35]	215.23	473.85	826.02	63.04	93.02
OneFormer (2023) [36]	372.15	775.05	1500.10	66.78	93.46
**NTSM (ours)**	**71.88**	**421.72**	**253.64**	**76.86**	**94.31**

**Fig. 7:**
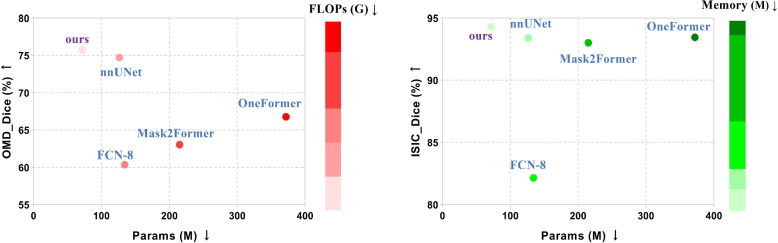
Params (M), FLOPs (G), Memory (M), and Dice results for different models. On the left, the X-axis represents Params (M) (the lower the better), the Y-axis represents OMD-Dice (%) (the higher the better), and the red represents FLOPs (G) (the shallower the better). On the right, the X-axis represents Params (M) (the lower the better), the Y-axis represents ISIC-Dice (%) (the higher the better), and the green represents Memory (M) (the shallower the better)

As shown in Fig. 7, we visualize the Params (M), FLOPs (G), Memory (M), and Dice results of each model for Table 2. The purpose is to demonstrate the relationship between model segmentation efficiency and performance. Our model has the lowest Params (M) compared to other large models, and it is 54.68M smaller than nnU-Net. Additionally, our model has the highest Dice score at 76.86%. This suggests that depthwise separable convolution in the FHPA module effectively reduces the number of model parameters and computations. Furthermore, the FHPA module is grouped and calculated along the channel dimension, which enhances the accuracy of model segmentation.

## Discussion

We conduct extensive ablation studies on the OMD dataset to validate the effectiveness of each module. To make the ablation results of each module more convincing, we use U-Net and nnU-Net as the backbones, and analyze them using sensitivity, specificity, 95HD, Dice, Params (M), FLOPs (G), and Memory (M) in both quantitative and qualitative manner.

### Ablation experiment (a): Verify the effectiveness of the modules in the non-salient target segmentation model (NTSM)

The NTSM mainly consists of a DA module that enhances non-salient feature differences and a FHPA module that reduces the number of parameters. We verify their importance by selecting different settings for each module. Table 3 shows the evaluation indicators of sensitivity, specificity, 95HD, Dice, Params (M), FLOPs (G), and Memory (M) after the ablation experiment (a). When we use U-Net as the backbone and join the DA module, we observe a increase of 17.84% in Dice and a decrease of 5.4mm in 95HD. After incorporating the FHPA module, the model's Params (M) decreases by 11.36M, FLOPs (G) decreases by 39.87G, and Memory (M) decreases by 43.22M. Moreover, when both modules are added, sensitivity, specificity, and Dice increase by 25.87%, 2.51%, and 18.31%, respectively. At the same time, 95HD, Params (M), FLOPs (G), and Memory (M) decrease by 6.65mm, 11.36M, 39.35G, and 43.17M, respectively. When nnU-Net is used as the backbone, the DA module increased Dice by 1.71% and decreased 95HD by 0.79mm. The FHPA module decreases Params (M) by 54.69M, FLOPs (G) by 51.27G, and Memory (M) by 100.21M. Enabling both modules simultaneously, results in a 0.19% sensitivity increase, a 0.06% in specificity increase, and a 3.14% Dice increase. Meanwhile, 95HD, Params (M), FLOPs (G), and Memory (M) decrease by 1.01mm, 54.68M, 44.51G, and 100.05M, respectively. The results indicate that the DA module increases the feature difference between lesions and non-lesions, significantly improving the segmentation performance of non-salient targets. In contrast, the FHPA module utilizes depthwise separable convolution to group learning, ensuring segmentation accuracy while effectively reducing the model's parameters and saving resource consumption.

**Table 3:** The results of ablation experiments for two modules on OMD dataset. The D + F represents the module as DA + FHPA

Backbone	Module	Sen (%)↑	Spe (%)↑	Dice (%)↑	95HD (mm)↓	Params (M)↓	FLOPs (G)↓	Memory (M)↓
U-Net	None	40.51	95.93	44.71	23.16	13.39	124.17	51.17
	DA	55.38	99.31	62.55	17.76	13.40	124.70	51.22
	FHPA	51.43	99.32	62.57	16.87	2.03	84.30	7.95
	D + F	66.38	98.44	63.02	16.51	2.03	84.82	8.00
nnU-Net	None	70.81	99.50	73.72	9.89	126.56	466.23	353.69
	DA	71.62	99.44	75.43	9.10	126.58	473.00	353.86
	FHPA	71.46	99.48	75.41	9.76	71.87	414.96	253.48
	D + F	71.00	99.56	76.86	8.88	71.88	421.72	253.64

**Fig. 8:**
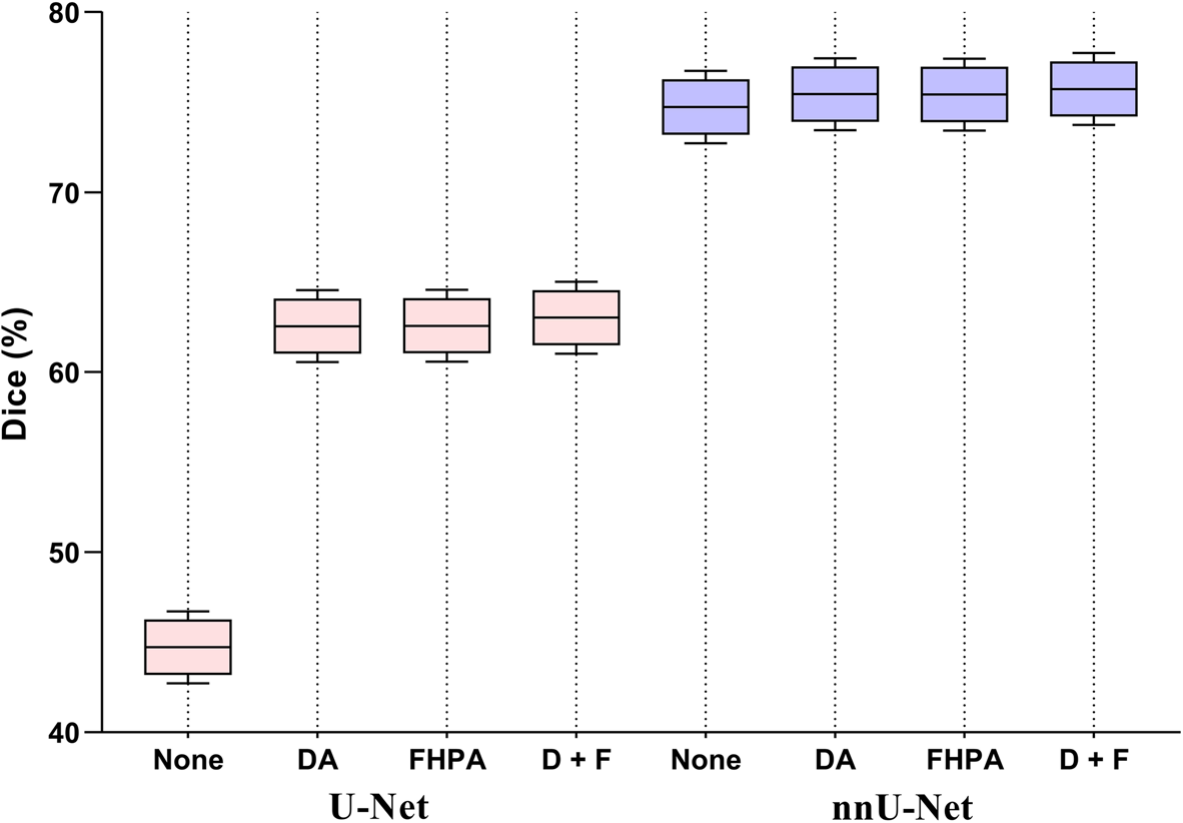
The five-fold cross-validation results of each model. The first to fourth columns show the Dice results of the five-fold cross-validation for each model when the backbone is U-Net, while the 5th to 8th columns show the Dice results of the five-fold cross-validation for each model when the backbone is nnU-Net. The D + F represents the module as DA + FHPA

**Fig. 9:**
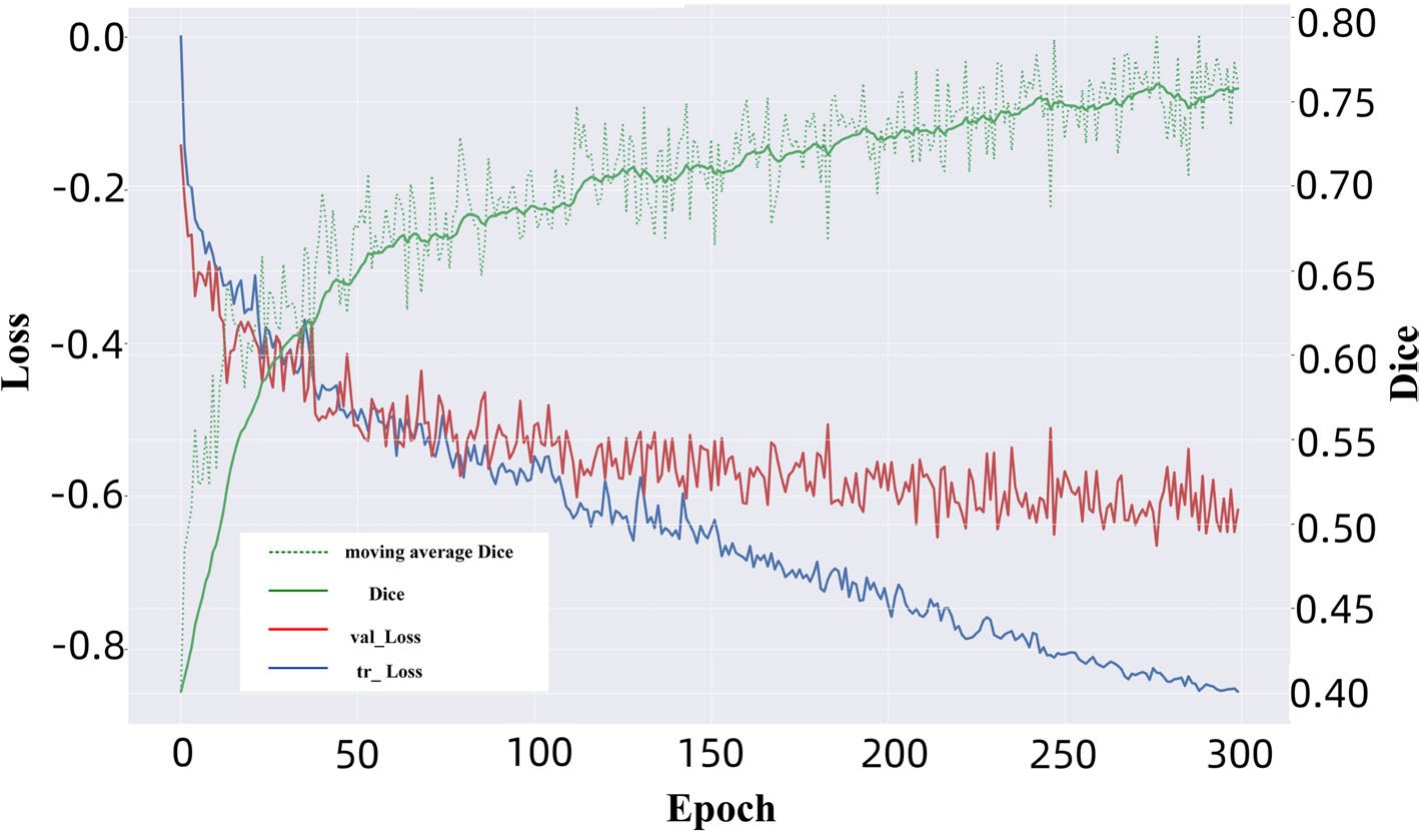
The changes in Loss and Dice during the training process of the non-salient target segmentation model (NTSM). The blue curve shows the change of Loss in the training set, while the red curve represents the change of Loss in the validation set. The green continuous curve represents the change of the moving average Dice, and the green discontinuous curve represents the change of the Dice

We adopt a five-fold cross-validation method to avoid experimental randomness. We randomly divide the training dataset into five equal parts, selecting four of them to participate in the actual training process each time, and the remaining one as the validation set. As shown in Fig. 8, the visualization results of the five-fold cross-validation of Dice for each model in Table 3 are presented. Through this approach, the randomness of a single evaluation is reduced, and the stability and reliability of the evaluation are improved. As shown in Fig. 9, the changes in Loss and Dice of the training and validation sets during the training process of the NTSM are shown. On the one hand, the Loss of the training set continues to decrease, while the Loss of the validation set first rapidly falls and then tends to stabilize. On the other hand, the Dice and moving average Dice during the training process both accelerate and stabilize. The result shows that the model fits normally and can learn effective lesions features to improve segmentation accuracy.

### Ablation experiment (b): Verify the effectiveness of the submodule of the DA module

The DA module mainly consists of two submodules, namely an LCD submodule that enhances low-level feature differences and an LSA submodule that enhances high-level feature differences. To determine the importance of each submodule, we conduct an ablation experiment by removing one submodule at a time. The experiment results, as shown in Table 4, include sensitivity, specificity, 95HD, and Dice evaluation indicators. When using U-Net as the backbone, adding the LCD submodule achieves a 11.2% increase in Dice, and a 2.6mm decrease in 95HD. Adding the LSA submodule resulted in a 12.59% increase in Dice and a 3.59mm decrease in 95HD. When both submodules are added simultaneously, Dice increases by 17.84%, and 95HD decreases by 5.4mm. On the other hand, when using nnU-Net as the backbone, and adding the LCD submodule, Dice increases by 1.51%, and 95HD decreases by 0.37mm. After adding the LSA submodule, Dice increases by 1.22%, and 95HD decreases by 0.11mm. Additionally, by adding both submodules simultaneously, Dice increases by 1.71%, and 95HD decreases by 0.79mm. The results indicate that the LCD submodule can effectively learn local and contextual information, while the LSA submodule can effectively learn logical semantic information. They enhance feature differences in non-salient lesions and improve segmentation performance.

**Table 4:** The results of ablation experiments for two submodules in the DA module on the OMD dataset

Backbone	Submodule	Sen (%)↑	Spe (%)↑	Dice (%)↑	95HD (mm)↓
U-Net	None	40.51	98.93	44.71	23.16
	LCD	54.25	98.79	55.91	20.56
	LSA	47.04	99.24	57.30	19.57
	DA (LCD + LSA)	55.38	99.31	62.55	17.76
nnU-Net	None	70.81	99.50	73.72	9.89
	LCD	71.70	99.52	75.23	9.52
	LSA	71.18	99.45	74.94	9.78
	DA (LCD + LSA)	71.62	99.44	75.43	9.10

**Fig. 10:**
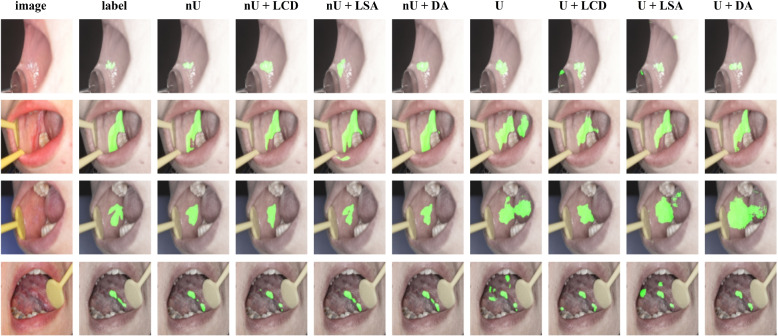
The change of images after adding different submodules. The nU represents the nnU-Net, and the U represents the U-Net. In the images, the green color denotes the segmentation areas

We intend to visually analyze how submodules contribute to the DA module. Figure 10 displays the segmentation changes of some images after entering different submodules. From the figure, we can observe that the LCD submodule can locate non-salient areas, while the LSA submodule focuses more on semantic features. Combining the two submodules improves the accuracy of the lesions range and enhances the interpretability of the model.

### Ablation experiment (c): Verify the effectiveness of the FHPA module

The number and location of FHPA modules have a significant impact on the number of parameters and computations required for image segmentation. To understand the importance of FHPA modules, we conduct an ablation experiment where we add one and three FHPA modules in the encoding phase and also add one FHPA module in the decoding phase. As shown in Table 5, we analyze the results in terms of Params (M), FLOPs (G), Memory (M), and Dice evaluation indicators. The results show that when using U-Net as the backbone, the addition of one FHPA module in the encoding phase resulted in a decrease of 4.38M Params (M), 3.59G FLOPs (G), and 16.68M Memory (M). After adding three FHPA modules to the encoding phase, Params (M) decreased by 8.62M, FLOPs (G) decreased by 27.9G, and Memory (M) decreased by 32.8M. Additionally, when we added one FHPA module to the decoding phase and three FHPA modules to the encoding phase, Params (M) decreased by 11.36M, FLOPs (G) decreased by 39.87G, Memory (M) decreased by 43.22M, and Dice increased by 17.86%. Similarly, when using nnU-Net as the backbone, the addition of one FHPA module in the encoding phase resulted in a decrease of 18.21M Params (M), 1.11G FLOPs (G), and 33.34M Memory (M). After adding three FHPA modules to the encoding phase, Params (M) decreased by 54.63M, FLOPs (G) decreased by 20.09G, and Memory (M) decreased by 100.02M. Moreover, adding one FHPA module to the decoding phase and three FHPA modules to the encoding phase resulted in a decrease of 54.69M Params (M), 51.27G FLOPs (G), and 100.21M Memory (M), and a 1.69% increase in Dice. These results suggest that adding more FHPA modules reduces the number of model parameters, but there is a limit to this reduction and a local optimal solution exists. Additionally, incorporating the FHPA module in the decoding phase improves segmentation performance by reducing the loss during feature recovery.

**Table 5:** The results of ablation experiments at different positions and numbers in the FHAP module on OMD dataset. Enc 1 indicates that the FHPA module is at the encoding position and has a quantity of 1, Enc 3 indicates that the FHPA module is at the encoding position and has a quantity of 3, and Dec 1 indicates that the FHPA module is at the decoding position and has a quantity of 1

Backbone	Module	Dice (%)↑	Params (M)↓	FLOPs (G)↓	Memory (M)↓
U-Net	None	44.71	13.39	124.17	51.17
	FHPA (Enc 1)	52.87	9.01	120.58	34.49
	FHPA (Enc 3)	57.95	4.77	96.27	18.37
	FHPA (Enc 3 + Dec 1)	62.57	2.03	84.30	7.95
nnU-Net	None	73.72	126.56	466.23	353.69
	FHPA (Enc 1)	74.28	108.35	465.12	320.35
	FHPA (Enc 3)	75.11	71.93	446.14	253.67
	FHPA (Enc 3 + Dec 1)	75.41	71.87	414.96	253.48

**Fig. 11:**
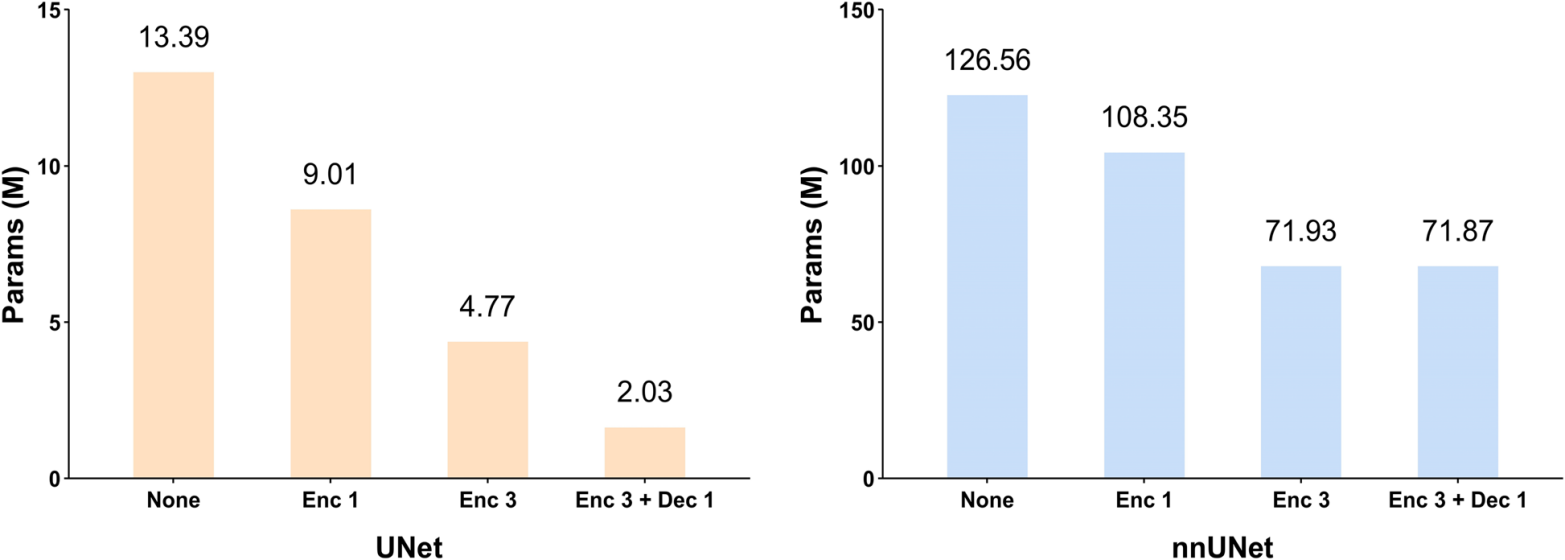
The variation of Params (M) using different models. The left side represents the changes in Params (M) for each model when the backbone is the U-Net, while the right side shows the changes in Params (M) for each model when the backbone is the nnU-Net. Different FHPA modules are used to represent different variations in the encoder and decoder stages of the models. The None indicates that the FHPA module has not been added, Enc 1 indicates that the FHPA module is at the encoding position and has a quantity of 1, Enc 3 indicates that the FHPA module is at the encoding position and has a quantity of 3, and Dec 1 indicates that the FHPA module is at the decoding position and has a quantity of 1

In Fig. 11, there are the variations of Params (M) for different models. Since the number of parameters varies with different backbones, the FHPA module has diverse effects in reducing the Params (M) of the model. When the backbone is U-Net, adding a single FHPA module reduces Params (M) by 4.38M. When the backbone is nnU-Net, adding a single FHPA module reduces Params (M) by 18.21M. By adding the FHPA module in the decoding part, the model can effectively improve segmentation performance while reducing Params (M). This ablation experiment demonstrates the reasonable use of the number and location of the FHPA module, which can improve segmentation performance and efficiency and meet the needs of mobile healthcare.

The FHPA module in our model is irreplaceable and we show this by conducting replacement experiments using the DGA module in MALUNet. The DGA module also reduces the number of parameters. Our results are presented in Table 6 with the Params (M), FLOPs (G), Memory (M), and Dice evaluation indicators. When using U-Net as the backbone, adding the DGA module, Params (M) decreased by 9.08M, FLOPs (G) decreased by 23.75G, Memory (M) decreased by 34.59M, and Dice increased by 13.46%. Replacing the corresponding position with the FHPA module, Params (M) decreased by 11.36M, FLOPs (G) decreased by 39.87G, Memory (M) decreased by 43.22M, and Dice increased by 17.86%. With nnU-Net as the backbone, adding the DGA module, Params (M) decreased by 48.65M, FLOPs (G) decreased by 32.14G, Memory (M) decreased by 77.28M, and Dice increased by 1.15%. After replacing the corresponding position with the FHPA module, Params (M) decreased by 54.69M, FLOPs (G) decreased by 51.27G, Memory (M) decreased by 100.21M, and Dice increased by 1.69%. Our results indicate that the FHPA module is better than the DGA module at reducing the number of model parameters and computations. The FHPA module achieves this through group learning with depthwise separable convolutions while still maintaining better segmentation performance.

**Table 6:** The results of ablation experiments for FHPA and DGA on OMD dataset

Backbone	Module	Dice (%)↑	Params (M)↓	FLOPs (G)↓	Memory (M)↓
U-Net	None	44.71	13.39	124.17	51.17
	DGA	58.17	4.31	100.42	16.58
	FHPA	62.57	2.03	84.30	7.95
nnU-Net	None	73.72	126.56	466.23	353.69
	DGA	74.87	77.91	434.09	276.41
	FHPA	75.41	71.87	414.96	253.48

## Conclusions

In this paper, we propose a non-salient target segmentation model (NTSM), which includes the DA module and the FHPA module. The DA module first learns low-level comparative information to extend segmentation masks to a wider range of potential regions. It then analyzes logical semantic relationship information to determine the true foreground and background. The FHPA module uses a new HPA mechanism to make self-attention’s quadratic complexity become linear, and it employs grouping to thoroughly grasp information from diverse perspectives, thus effectively cutting down on parameters. Experiments show that our model achieves much higher segmentation accuracy in non-salient target compared to other methods, and it can significantly decrease resource demands. Compared to the nnU-Net model, our model achieved a 3.14% increase in the dice index while decreasing the number of model parameters by 54.68M. However, our model has certain limitations. Due to the large number of parameters in nnU-Net, resulting in our model obtains the optimal solution, but is still insufficient for some lightweight models. We hope our work can inspire the further application of artificial intelligence in the medical field. While ensuring segmentation accuracy, delve deeper into or design better backbones.

## Data Availability

The datasets generated and analysed during the current study are not publicly available due to privacy of participants but are available from the corresponding author on reasonable request.

## Abbreviations

NTSM: Non-salient target segmentation model
DA: Difference association
FHPA: Feature hierarchy pyramid attention
HPA: Hadamard product attention
OMD: Oral mucosal diseases
ISIC: International skin imaging collaboration
CNNs: Convolutional neural networks
LCD: Local context difference
LSA: Logical semantic association
LCE: Low-level comparison extractors
LFE: Low-level feature extractors
LR: Local receptor
CR: Context receptor
BI: Bilinear interpolation
DW: Depthwise separable convolution
HP: Hadamard product
Sen: Sensitivity
Spe: Specificity
Dice: Dice similarity coefficient
95HD: 95% Hausdorff distance
SOTA: State-of-the-art
